# Vaccination in the Light of the Royal British Commission

**Published:** 1899-03

**Authors:** M. R. Leverson

**Affiliations:** Fort Hamilton, L. I., N. Y.


### VACCINATION IN THE LIGHT OF THE ROYAL BRITISH COMMISSION.

M. R. LEVERSON, M. D., FORT HAMILTON, L. I., N. Y.

(Continued from Jan. No., page 22.)

*The Pall Mall Gazette*, of February 21st, 1890, says: "The news is confirmed that leprosy is spreading in New Caledonia, and about 3,000 aborigines had been attacked. Two lazar-houses have recently been built."

"On June 2d Mr. Gourlay called the attention of the Under-secretary for the Colonies to the increase of leprosy in Jamaica. Mr. H. Brown, editor of *The Simla Herald*, writes from Simla on October 2d, 1889, 'Leprosy is spreading almost by leaps and bounds.' In a lecture before the Bath Literary and Philosophical Association, January, 1890, Mr. Austin T. King quoted statistics from the census returns in India, showing that in 1872 there were 99,073 lepers. In the census returns of 1881 the numbers were put at 131,618."

Q. 10,162 (to Sir Charles Dalrymple). The answer of the Under-secretary to the question put to him by Mr. Gourlay was, "There is a considerable number of lepers in certain districts in Jamaica, but the Secretary of State is not aware whether the number is increasing."

Although not forming part of Mr. Tebb's testimony, it seems to the editor that it will be of use to the student, whether of leprosy or of vaccination, to give here the summary of the conclusions arrived at by Mr. Tebb, in his work, *The Recrudescence of Leprosy*. It is true that Mr. Tebb is not a doctor of medicine but it is the opinion of the editor that he is a man of accurate observation and of painfully conscientious reasoning. He decidedly knows more about leprosy and vaccination than any physician the editor has ever known. The summary now given will be found on pages 350-2, of the work above named:

"1. That leprosy has greatly increased during the last half century, and that it is prevalent in many places where it was formerly unknown.

"2. That whilst the opinion of medical authorities and experts varies considerably on the subject of the contagiousness of leprosy, the preponderance of authority is in favor of the theory that it is not contagious in the ordinary sense of the term, but is communicable by means of a cut, sore, or abraded surface; and this view is confirmed by my own personal investigations.

"3. That other alleged factors such as malaria, a fish diet, syphilitic cachexia, heredity, and insanitation are admittedly unequal to explain the rapid growth of the disease in certain of our (the British) colonies and dependencies, as well as in other countries.

"4. That on one point there is much agreement and hardly any dissent, viz.: the inoculability of leprosy, and that the view of leprosy as an inoculable disease, while it is most clear to those who take the malady to be due to a bacillus, is older than the bacteriological evidence and is not dependent thereon.

"5. That the most frequent opportunities of inoculating the virus of leprosy are afforded in the practice of inoculating vaccine, which is the only inoculation that is habitual and imposed by law; and that the evidence here adduced is calculated to show that vaccination is a true cause of the diffusion of leprosy.

"6. That the official information, collected by interrogatories and otherwise, has not been hitherto of a kind to show how far vaccination has determined the amount of leprosy in recent times, and that any interrogatories that may be sent out in future should not be limited to ascertaining the effects, as regards leprosy, of hypothetically 'pure' lymph When on very rare occasions interrogatories have been submitted they have been framed to ascertain the results of a purely hypothetical system of vaccination, which is not anywhere discoverable in practice, and alleged to be unattainable (*i. e.*, with pure lymph, and free from hereditary taint), and the replies are therefore futile and misleading.

"7. That with the exception of two groups of cases, those adduced by Dr. Roger S. Chew, of Calcutta, and Dr. S. P. Impey, of Robben Island, those reported in this volume (*The Recrudescence of Leprosy*) have not been the result of special investigation, but have cropped up accidentally in the course of medical practice, and in some instances have been published by practitioners with apologies to the profession for presenting such unwelcome disclosures.

"8. That the increase of leprosy in the Sandwich Islands, the West Indies, the United States of Colombia, British Guiana, South Africa, and New Caledonia has followed, *pari passu*, with the introduction and extension of vaccination, which, in nearly all these places, without previous inquiry or demand from the inhabitants, has been made compulsory.

"9. That as leprosy is a disease of slow incubation, often taking years to declare itself, and in its incipient stages can be detected only by practitioners of large experience, it follows that in countries where leprosy exists there is great danger of extending the disease by arm-to-arm vaccination.

"10. Leprosy being one of the most loathsome diseases to which the human race is subject, and being practically incurable, it behooves all interested in the public well-being to do their best to prevent its diffusion, and, as a means thereto, to discourage the practice of vaccination on that ground, if on no other."*

Q. 10,165. Mr. Tebb quotes the report of the Select Committee of Victoria upon the vaccination law, together with the proceedings of the committee, minutes of evidence, and appendices ordered by the Legislature to be printed March 24th, 1881:

"Fourteen witnesses were examined, of whom six were public vaccinators, and six leading metropolitan medical practitioners. A list of questions was also transmitted to prominent medical men in Ballarat, Geelong, and Sandhurst; and the replies received from them appear in a tabulated form in Appendix B.

"Your committee find that the opinions expressed by the various medical men are so conflicting and contradictory on

---

* The use of the so-called calf lymph, common in the United States, and now proposed in England, is but another of those "shuffles," whereof, Wm. Cobbett, referring to Jenner, said "Quackery has always one shuffle left." Small-pox poison passed through the cow until from twenty to thirty ulcers are formed, from which the poisonous matter mixed with the animal's blood is taken wherewith to impregnate the blood of healthy human beings, is not only likely to communicate small-pox to the vaccinee, but strange to say, ulcers on the cow, from whatever source, seem to have a tendency to take on a form or character resembling syphilis. It will also communicate tuberculosis if the vaccinifer cow be tuberculous, a disease to which the bovine race is specially disposed. Many kinds of skin diseases are also liable to be inoculated, though they may not have been recognized in the cow at the time. The long continuance of the process of milking, long after a calf would have been driven off by the cow, which is generally practised from evident economic reasons is probably the main cause for the tubercular disposition of the bovine race.—M. R. L.

many points as to render their testimony of little practical value.

"On the question of re-vaccination, for example, some stated that it was not necessary; others that it should take place every seven years, whilst others, again, would extend the period to 14 years. There was the same diversity of opinion with regard to the number of punctures or incisions to be made in the arm. One witness affirmed that the operation would be of little efficacy unless four punctures were made, and suggested that all vaccinators should be compelled to make that number. Another witness considered one cicatrix sufficient, and a third thought it would be better left to the discretion of the medical man, as some children were not strong enough to bear four marks." . . . Greater unanimity prevailed on the question of the communication of extraneous diseases, such as syphilis and scrofula, by vaccination, although some of the witnesses maintained that there would be no liability to such transmission unless blood were drawn during the operation. Dr. Beaney and Dr. Sparling, however, mentioned instances that came under their observation, of syphilis and erysipelas being communicated to children from purely colorless vaccine matter which contained no trace of blood." *

Qs. 10,166-7. There was also a committee which reported to the parliament in New South Wales, and in view of the conflict of testimony there also found, legislation was deemed inappropriate, and there is no compulsory law in that colony.

Q. 10,169. In the New South Wales report Dr. Jean Werner Gunst testified that when he was exploring in Madagascar he vaccinated different tribes and found it introduced a number of new diseases. It was the same in New Caledonia.

---

* Strange to say, after expressing these views, the committee contents itself with reporting a recommendation, that calf "lymph," which always contains blood, be substituted for "arm-to-arm"!—M. R. L.

Q. 10,170. Dr. Gunst states: "I was for seven years the only doctor for 120 miles at the Richmond and Clarence Rivers, New South Wales, and I had the best opportunity to watch the effect of vaccination. I was not like a public vaccinator who takes the vaccine and sees no more of the children, but I had to follow up the children and see if they became ill and if they were ill they came to me. I have seen scrofula and eczema and skin diseases of different types, which never appeared in the family before, attack children that before were healthy, and from my experience I calculated that in about 20 per cent. of cases of arm-to-arm vaccination diseases are produced in a family which were never known in it before."

Q. 10,170. In *The Pall Mall Gazette* for October, 1872, is an article under the heading "Vaccination on Virgin Soil," relating to the resistance to introduction of compulsory vaccination in the Isle of Foula (one of the Shetlands). Mr. Tebb wrote to the Registrar of the Island for information, on April 7th, 1883, and about a year later received the following reply:

"Dear Sir:—Your letter of April 7th, 1883, only reached me lately. There have been no cases of small-pox in our Isle, now, for fifty years back. The parish doctor visits us every two years to vaccinate the children, and many of the parents refuse to permit the operation, because we have seen so much damage done to children through vaccination. The Island numbers in all 267 inhabitants, young and old. Many of the children are being destroyed through skin eruptions, and, instead of being healthy, as formerly, are now sickly and yellow, and some have never had a day's health, and all through vaccination.

"Peter Peterson,

"*Registrar of Foula Isle.*"

Wishing for later information, on July 18th, 1889, Mr. Tebb wrote again and received the following reply:

FOULA ISLE, AUGUST 26th, 1889.

"WM. TEBB, ESQ.,

"DEAR SIR:—Yours of July 18th is to hand, on vaccination.

"First, no small-pox ever visited this Isle since year 1800.

"Second, the effect of vaccination upon the public health has been very bad.

"Third, there has been increase of children's diseases, caused by vaccination.

"Fourth, the present population of the Island is 264.

"Fifth, the people all object to vaccination, and many of the parents of children will rather suffer the whip of the law than give up their children to be vaccinated."

Qs. 10,173-4. Gives a quotation from the work on *Constitutional Syphilis,* by Dr. James George Beaney, surgeon and teacher of practical and operative surgery to the Melbourne Hospital, expressing a positive opinion that "syphilis is in very many instances communicated by means of 'child's vaccine lymph.' "

Q. 10,176. Refers to the Elberfeld disaster in 1887. Report received from the burgomaster of Elberfeld:

"Report on the skin diseases which prevailed during the summer, in consequence of vaccination with animal lymph.

"A few children were taken ill at the end of April of the present year (1887) and several hundred more at the end of June, in connection with vaccination with the animal vaccinating paste supplied by me, presenting symptoms which, for the present, I may briefly explain by stating that they coincide completely with those observed in the summer of 1885, at Wittow, on the Island of Rügen, and in the district of Cleve, which were described at the time as *impetigo contagiosa* (or moist tetter). During the nine years of the existence of my establishment these were the first complications arising from animal vaccination brought to my cognizance.

"There were, in all, three calves to which the vaccine in question could be traced back."

Then, after making various efforts to show that there was no "causal nexus" between the sickness of the children and one of the calves, he admits that from 600 to 800 children were taken ill, and not only does not deny but expressly admits that "there was no further doubt entertained as to the connection of the latter with the vaccine employed." On page 4 he says, "The vaccinating lymph was derived from the vaccinating institute of Stettin. It had originally been taken from two children at Füllchad, near Stettin, and was diluted with a solution of Thymol (1 : 1000) in the proportion of 2 (lymph) 1 (Thymol diluted)," and on page 5 he says, "It was not possible to find out the nature of the defect in the lymph."

Q. 10,188 (Mr. Hutchinson). "You are aware that impetigo contagiosa is a very slight ailment?" I am aware that it is a very troublesome disease."

Qs. 10,192-3. Shows a statement, *Lancet*, July 17th, 1880, by Mr. J. T. Hibbert, parliamentary secretary of the Local Government Board and a member of the Select Committee on Vaccination which sat in 1871, in which he admitted that the increase in infantile syphilis from 255, in 1847, to 1,554, in 1875, was largely due to vaccination; also the denial of the same by the editor of *The Lancet.*

Qs. 10,195-10,200. In reply to Mr. Hutchinson, Mr. Tebb says he does contend that this increase of infantile syphilis is due to vaccination, and Mr. Hutchinson questions him as to Ireland, but he declines to go into the Irish statistics at present, or to say anything more about Ireland except that "the Irish are an extremely moral race."

Q. 10,203. Return No. 443, entitled, Vaccination Mortality, dated 1877, page 15: Deaths from syphilis in 1847, under one year, are given at 255, increasing to 1,554 in 1875. In a later return, No. 392, dated August, 1880, the deaths from syphilis, under one year, per 1,000,000 of births in 1847, are 472, and there is a gradual increase until the last date given in the return, viz.: 1878, when it reaches 1,851 per 1,000,000 births.

Q. 10,204. Not being a medical man Mr. Tebb does not feel himself qualified to take a scientific view of the whole case, so he puts the statistics before the committee and proposes to quote Mr. Brudenell Carter to show their connection with vaccination.

Q. 10,205. Mr. Brudenell Carter says in *The Medical Examiner* of May 24th, 1877, "I think that syphilitic contamination by vaccine lymph is by no means an unusual occurrence, and that it is very generally overlooked because people do not know when or where to look for it. I think that a large proportion of the cases of apparently inherited syphilis are in reality vaccinal, and that the syphilis in these cases does not show itself until the age of from eight to ten years, by which time the relation between cause and effect is apt to be lost sight of." And in his treatise upon diseases of the eye, page 276, he says: "I have, myself, strong conviction that the physical peculiarities and proclivities which are usually due to syphilitic parentage are sometimes produced, even in an exaggerated form, by vaccination with lymph, yielded by a vaccinifer who has himself inherited the disease, and that this may happen without the production of any derangement of the course of the vaccine vesicle, or of anything which could, at the time, be recognized as evidence of a specific inoculation."

Q. 10,215. In reply to Mr. Hutchinson Mr. Tebb says he will look up the facts to see if he can find any record in the official department of a case of syphilis during the 20 years in which Mr. Hart asserted there had been none. He then quotes *Lecons sur la Syphilis Vaccinale*, by Mr. Fournier, who holds to-day the same high position as was formerly held by Prof. Ricord, pages 3, 4. From a lecture to medical students, the subject is, Vaccinal syphilis not to be mentioned in public nor "anywhere else but in this amphitheatre," because of its tendency to do disservice to vaccination. Then, page 6, the subject is, "The Rarity of Vaccinal Syphilis." "Is vaccinal syphilis quite authenticated? Is it absolutely demonstrated in spite of the denials which have been opposed to it? Is it in a word definitely established that a sound subject may receive syphilis by the intermediary of a vaccine lymph taken from a syphilitic source? * Yes, a hundred times yes; and it is the demonstration of that fact which must occupy us to begin with." On pages 6-12 the lecturer treats of the case of Mr. Millard's patient syphilized by vaccine lymph furnished by the Academy of Medicine. Then on pages 12-17 he relates in detail the case of Dr. Cory, and on pages 17, 18 concludes that there is a real and serious danger, and as every individual is destined to undergo once or several times in his life the vaccine inoculation, the danger of vaccinal syphilis is encountered by all the world once or several times in the course of his life, and is particularly grave in the young, often terminating fatally. Pages 19-30 explains how vaccine syphilis may be propagated and become a public danger; and then on page 53 we have a lecture on the subject of "Vaccinal Syphilis is not to be Disclosed;" that states that there are many more cases on the cards or in the memories of practitioners than in the columns of our journals; that he him-

---

*It is to be borne in mind that before vaccine matter is taken from the arm of a child even by a not very scrupulous operator, care has nearly always been taken to select a robust and perfectly healthy appearing child.

To accuse that child of having inherited syphilis when syphilis breaks out among those vaccinated from it, or even when it afterwards shows itself in the vaccinifer, is without foundation either in logic or pathology. Had not the profession been blinded by their professional religion they would have recognized the fact that they had themselves given the syphilis to the vaccinifer, and that its parents and itself were alike guiltless, until the vaccinator inoculated syphilis into the vaccinifer by their "vaccination," even though performed with the "pure calf lymph" of the New York Board of Health.

self had knowledge of two actual epidemics of vaccinal syphilis which have been kept secret and hushed up.

Qs. 10,216-20. The chairman seeks to weaken the effect of the foregoing statements by Prof. Fournier, as to the concealment of the facts, by the profession, "in the interest of vaccination."

Q. 10,222. (To Dr. Collins) the quotations from Prof. Fournier's work are given in order to contest the statement that "the alleged injury arising from vaccination is indeed disproved by all medical experience."

Q. 10,224 (Mr. Hutchinson). "It is universally admitted that syphilis may be transmitted by vaccination?" "Yes; whereas up to 1860 it was universally denied and has been officially denied in England much more recently."

Qs. 10,230-1. In reply to Mr. Picton, and referring again to Mr. Hart's statement (quoted supra, Q. 9,971), Mr. Hutchinson testified before the committee of the House of Commons that the medical department of the Local Government Board had themselves communicated to him facts of the kind (viz.: of vaccinal syphilis).

Q. 10,237-40. So far from such cases being due to "gross and culpable carelessness on the part of the vaccinator," Mr. Fournier says: "With the utmose care accidents cannot be avoided." Also, that "syphilis may be communicated where there is no trace of blood" (*i. e.*, in the vaccine virus itself.—M. R. L.). The same conclusion was arrived at "upon the investigation of a well-known case of invaccination of syphilis which happened to an official in this country."*

Q. 10,241. Ricord, Chaumel, Moreau, Rostan, Velpean, and others, who in 1871 alleged that they did not know of any case of syphilis induced by vaccination,† have changed their opinions.

---

* Presumably the case of Dr. Cory.—Ed.

† Report of Committee of 1871, Q. 505.

